# Mortality and readmission rates of patients discharged in-hours and out-of-hours from a British ICU over a 3-year period

**DOI:** 10.1038/s41598-022-10613-1

**Published:** 2022-04-22

**Authors:** Julian Cumberworth, Mandy Chequers, Stephen Bremner, Owen Boyd, Barbara Philips

**Affiliations:** 1 Department of Intensive Care Medicine, Royal Sussex County Hospital, University Hospitals Sussex NHS Foundation Trust, Brighton, BN2 5BE UK; 2 Brighton and Sussex Medical School, University of Sussex, Brighton, BN1 9PX UK

**Keywords:** Outcomes research, Health care, Medical research

## Abstract

Excess in-hospital mortality following out-of-hours ICU discharge has been reported worldwide. From preliminary data, we observed that magnitude of difference may be reduced when patients discharged for end-of-life care or organ donation are excluded. We speculated that these patients may be disproportionately discharged out-of-hours, biasing results. We now compare in-hospital mortality and ICU readmission rates following discharge in-hours and out-of-hours over 3 years, excluding discharges for organ donation or end-of-life care. This single-centre retrospective study includes patients discharged alive following ICU admission between 01/07/2015–31/07/2018, excluding readmissions and discharges for end-of-life care/organ donation. A multiple logistic regression model was fitted to estimate adjusted odds ratio of death following out-of-hours versus in-hours discharge. Characteristics and outcomes for both groups were compared. 4678 patients were included. Patients discharged out-of-hours were older (62 vs 59 years, p < 0.001), with greater APACHE II scores (15.7 vs 14.4, p < 0.001), length of ICU stay (3.25 vs 3.00 days, p = 0.01) and delays to ICU discharge (736 vs 489 min, p < 0.001). No difference was observed in mortality (4.6% vs 3.7%, p = 0.25) or readmission rate (4.1% vs 4.2%, p = 0.85). In the multiple logistic regression model out-of-hours discharge was not associated with in-hospital mortality (OR = 1.017, 95% CI 0.682–1.518, p = 0.93). Our findings present a possible explanation for reported excess mortality following out-of-hours ICU discharge, related to inclusion of organ donation and end-of-life care patients in data sets rather than standards of care delivered out-of-hours. We are not aware of any other studies investigating the influence of this group on reported post-ICU mortality rates.

## Introduction

Out-of-hours ICU discharge has been independently associated with in-hospital mortality in studies from Australia^1–5^, Canada^6–8^, the UK^9,10^, and France^11^. Two recent meta-analyses report odds ratios of 1.31 (95% CI 1.25–1.38) and 1.39 (95% CI 1.24–1.57) for death following out-of-hours ICU discharge compared to in-hours ICU discharge^12,13^. Concern around out of hours discharges is emphasised in the UK by the NHS service specification for Adult Critical Care stating that transfer from Critical Care to a ward must occur between the hours of 07:00 and 21:59, and ideally between 07:00 and 19:59^14^.

Heterogeneous ICU populations, differing healthcare systems and varying out-of-hours definitions make direct comparisons between studies challenging. Not all studies have demonstrated an association between out-of-hours ICU discharge and in-hospital mortality^15–18^. We have previously investigated outcomes following discharge from our ICU in-hours and out-of-hours over a 6 month period, and reported higher mortality rates in patients discharged from the ICU out-of-hours than in-hours^19^. However, in this small sample, the difference narrowed when patients discharged for organ donation or end-of-life-care were excluded. A disproportionate number of end-of-life patients were discharged out of hours and since the mortality of these patients is 100% even small numbers may skew mortality rates. In this much larger single-centre study we investigate this further, comparing mortality rates between those discharged in-hours and out-of-hours, before and after exclusion of those discharged for end-of-life care or organ donation, over a 3-year period in the Intensive Care Units of a single hospital.

## Methods

This is a single centre retrospective study based on anonymised data from our intensive care unit database. We investigated in-hospital mortality following ICU discharge in-hours and out-of-hours, after identification and exclusion of patients discharged for end-of-life care or organ donation. The requirement for ethical approval was checked for using Health Research Authority (HRA) approval decision tool and the requirement for ethics waived by the University Hospitals Sussex NHS Foundation Trust R&D department. The study was conducted accordance with relevant guidelines and regulations including the Declaration of Helsinki.

The Royal Sussex County Hospital, UK, is a Major Trauma Centre and tertiary centre for a number of medical and surgical specialties with a diverse case mix. ICU services comprise a mixed medical and surgical adult ICU and neurosurgical ICU with a total of 19 level three and 12 level two beds (see “Definitions”). There is 24 h consultant cover which is on-site from 08:00 until late in the evening. At night the on-site medical cover includes a registrar and two senior house officers (residents), from 20:30 to 08:30. A nurse-led critical care outreach team is available from 07:30 until 20:00. The ICU and ward team nursing shift patterns are 12 h shifts, with handovers at 07:30 and 19:30.

All admissions from July 2015 to July 2018 were identified from the local ICU databases (Metavision Suite, iMDSoft, Tel Aviv, Israel; Ward Watcher v13.07, Critical Care Audit Ltd). Patients who did not survive to ICU discharge were excluded. Readmissions (defined as readmission to ICU within the same hospital stay) were identified by detecting multiple listed ICU admissions with the same hospital admission date for the same patient. For these patients, medical records were obtained and checked to clarify admission status. This also enabled readmission rates to be determined. In the overall analysis of time of discharge, the first ICU admission per hospital stay was retained within the data set, and readmissions excluded. Patients with an APACHE II score recorded as 0 were excluded (primarily those with length of ICU stay < 8 h).

Organ donors were identified by cross-checking local organ donation records with ICU discharges. Patients discharged for end-of-life care were identified from the existing ICU admissions database. These groups were excluded, leaving the final study sample.

Data collected included age, sex, hospital admission time and date, ICU admission time and date, length of ICU stay, ICU discharge time and date, length of stay post ICU, hospital discharge date, outcome, ventilator days, days of level 2 or level 3 support, APACHE II score at ICU admission, nature of admission (medical or surgical), and whether an admission was a readmission.

### Definitions

The in-hours period is defined as 07:01 to 21:59 and the out-of-hours period as 22:00 to 07:00, as used previously^19^. A readmission is defined as readmission to ICU within the same hospital stay. End-of-life-care describes patients with reason for discharge recorded as ‘palliative care’. In-hospital mortality after ICU discharge describes mortality at any point after discharge following a patient’s first ICU admission, prior to hospital discharge. Length of stay following ICU discharge therefore describes the time period between ICU discharge and death or hospital discharge.

Level 2 describes patients requiring more detailed observation or intervention including support for a single failing organ system or post-operative care and those stepping down from higher levels of care. Level 3 describes patients requiring advanced respiratory support alone or monitoring and support for two or more organ systems^20^.

### Statistical analysis

ICU medical records were reviewed using iMDsoft^®^ Ltd MetaVision ICU software. Microsoft Excel was used to review the initial data set prior to statistical analysis, which were performed using IBM^®^ SPSS Statistics 25 and Stata^®^ 15 software^21^.

Data for the previously described variables were compared, for the patients discharged in-hours and out-of-hours, by Chi-squared or Mann–Whitney *U* tests as appropriate. A multiple logistic regression model was developed including baseline patient characteristics and those where statistical comparisons between the in-hours and out-of-hours groups for which a test of difference was significant at the 5% level. The model included APACHE II score, in-hours vs out-of-hours discharge, nature of admission (medical vs surgical), sex, length of ICU stay, time between hospital and ICU admission, whether ventilatory support was required and discharge delay (time between decision to discharge from ICU and discharge).

## Results

**Figure 1:**
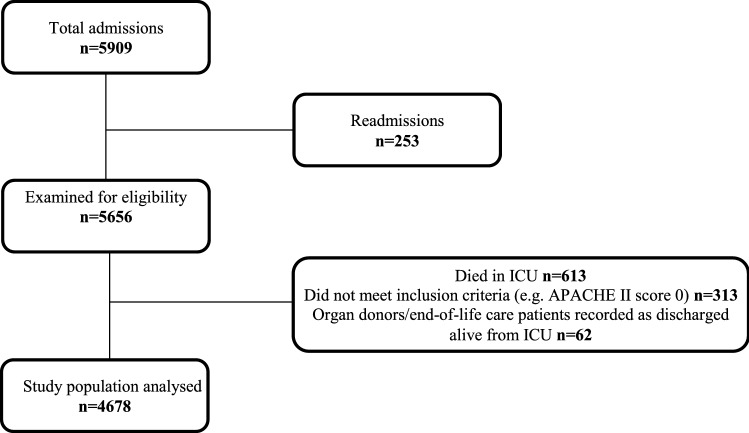
Flowchart describing formation of final study population.

A total of 5909 patients were considered (Fig. 1). After all exclusions were applied 4678 ICU admissions remained, of whom 3943 (84.3%) were discharged from the ICU in-hours, and 735 (15.7%) out-of-hours. Patient characteristics are described in Table 1. Patients discharged within hours were younger than patients discharged out of hours (59 versus 62 years, p < 0.001) and the admission APACHE II score was lower in those discharged within hours than out-of-hours (14.40 versus 15.74, p < 0.001).

**Table 1:** Characteristics of patients discharged from the intensive care unit (ICU) after exclusion of end-of-life patients, over a 3-year period.

Characteristic	In-hours discharge	Out-of-hours discharge	*P* value	Total
*N*	3943	735	n/a	4678
Overall % of sample	84.29	15.71		100
**Sex**				
Male	2260 (83.8%)	436 (16.2%)	0.31	2696
Female	1683 (84.9%)	299 (15.1%)		1982
Mean age [95% CI], SD (years)	59.16 [58.62–59.70], 17.44	62.00 [60.78–63.22], 16.87	< 0.001	59.61 [59.11–60.10], 17.38
Mean APACHE II score [95% CI], SD	14.40 [14.22–14.58], 5.79	15.74 [15.31–16.17], 5.88	< 0.001	14.61 [14.44–14.78], 5.82
Median length of stay in ICU, IQR (days)*	3.00 (1.67–6.04)	3.25 (1.89–6.09)	0.01	3.04 (1.69–6.05)
Median length of stay post ICU, IQR (days)	6 (2–13)	7 (3–15)	< 0.001	6 (2–13)
Median delay to discharge, IQR (min)	489 (205–1835)	736 (250–2168)	< 0.001	529 (210–1861)
Median length of level 2 support, IQR (days)	2 (2–4)	2 (2–4)	0.87	2 (2–4)
Median length of level 3 support, IQR (days)	0 (0–2)	0 (0–2)	0.51	0 (0–2)
Level 3 support required	1367 (34.67%)	266 (36.19%)	0.43	1633 (34.91%)
Median length of ventilatory support (days)	0 (0–2)	0 (0–2)	0.92	0 (0–2)
Ventilatory support required	1220 (30.94%)	230 (31.29%)	0.85	1470 (31.42%)
**Nature of admission (% medical)**	47.05	51.02		47.67
Medical	1855	375	0.05	2230
Surgical	2088	360		2448
* Of which elective*	1029	141		1170
* Of which non-elective*	1059	219		1278
**ICU readmission rate (%)**	4.24	4.08		4.21
Readmitted	167	30	0.85	197
Not readmitted	3776	705		4481
**Post-ICU mortality rate (%)**	3.73	4.63		3.87
Died	147	34	0.25	181
Survived	3796	701		4497

*Data for this variable recorded to 2 decimal places. Data compared by Mann–Whitney *U* or Chi-square tests as appropriate.

The overall median length of stay in ICU was 3.04 (1.69–6.05) days. In patients discharged out-of-hours the length of ICU stay, the length of hospital stay post-ICU (endpoint defined as death or hospital discharge), and the delay to ICU discharge were longer than patients discharged within hours (Table 1). The median length of level 2 support was 2 (2–4) days overall. Median lengths of level 2 and level 3 support were comparable in the in-hours and out-of-hours groups. 34.7% of patients required level 3 support (defined as length of level 3 support ≥ 1 day) in the in-hours discharge group, and 36.2% in the out-of-hours group. 31.4% of patients required ventilatory support (defined as length of ventilatory support ≥ 1 day), and this proportion was similar across both groups. There was no difference in the median length of time on ventilatory support.

Overall 2230 (47.7%) of patients were medical admissions and 2448 (52.3%) were surgical admissions, of which 1170 (47.8%) were elective and 1278 (52.2%) were urgent or emergency surgery. Out-of-hours, more medical than surgical patients (51.0% vs 49.0%) were discharged, whilst within hours the majority of patients were surgical patients (53.0%). The group of surgical patients discharged in-hours were more likely to be planned elective patients than the surgical patients discharged out-of-hours (49.3% vs 39.2%).

One hundred and ninety-seven patients (4.21%) were readmitted. Readmission rates were comparable for those discharged in-hours and out-of-hours (4.24% and 4.08% respectively). 181 patients died in hospital following ICU discharge, giving an overall post-ICU in-hospital mortality rate of 3.87%. There was no observed difference in hospital mortality rates following discharge in-hours or out-of-hours (3.73% vs 4.63%, p = 0.25) as shown in Table 1.

By contrast, the overall post-ICU mortality rate *before* exclusion of those recorded as surviving to ICU discharge, but discharged for organ donation, was 4.89% (4.56% following in-hours discharge and 6.65% following out-of-hours discharge, p = 0.02). The ICU readmission rate was 4.18% (3.99% following in-hours discharge and 4.21% following out-of-hours discharge, p = 0.08).

A multiple logistic regression model for patient-related factors, ICU-related factors and the likelihood of post-ICU in-hospital mortality was formulated and results are reported in Table 2. The model did not demonstrate an association between out-of-hours ICU discharge and mortality (OR = 1.017, 95% CI 0.682–1.518). Variables independently associated with mortality following ICU discharge were APACHE II score (OR 1.122, 95% CI 1.095–1.148), length of ICU stay (OR 1.018, 95% CI 1.002–1.035), and time from hospital to ICU admission (OR 1.047, 95% CI 1.030–1.064).

**Table 2:** Multiple logistic regression model showing associations between predictor variables and the outcome variable of in-hospital mortality, in patients discharged alive from our intensive care unit (ICU) over a 3-year period (n = 4678).

Variable	Odds ratio	95% confidence interval	P value
APACHE II score (per point increase)	1.122	1.095–1.148	< 0.001
Out-of-hours discharge	1.017	0.682–1.518	0.93
Medical admission	1.344	0.959–1.883	0.09
Female sex vs male	0.827	0.602–1.138	0.24
Days between hospital admission and ICU admission (per day increase)	1.047	1.030–1.064	< 0.001
Length of ICU stay (per day increase)	1.018	1.002–1.035	0.03
Ventilatory support required	1.265	0.899–1.780	0.18
Delay from request to discharge (per hour)	1.000	0.998–1.002	0.92

## Discussion

We report no clinically significant difference in in-hospital mortality rate between patients discharged in-hours and out-of-hours from the ICU, after exclusion of those discharged for organ donation or end-of-life care (mortality 3.73% following in-hours discharge vs 4.63% out-of-hours, p = 0.25), and multiple logistic regression showed that out-of-hours discharge was not associated with increased likelihood of mortality in this dataset (OR = 1.017, 95% CI 0.682–1.518). By contrast, a significant difference had been demonstrated when these patients were included (mortality 4.56% following in-hours discharge vs 6.65% out-of-hours, p = 0.02). We also report no clinically significant difference in readmission rate (4.24% following in-hours discharge vs 4.08% out-of-hours, p = 0.85). To our knowledge, previous studies investigating mortality and morbidity in patients discharged out-of-hours from ICU have not explored the possible influence of such patients. We theorise that excluding patients discharged alive from ICU for organ donation or end-of-life care leaves a cohort more representative of the average patient discharged from the ICU following clinical improvement, for ongoing recuperation. Whilst the majority of previous research in this field has demonstrated excess mortality in those discharged from the ICU out-of-hours, the influence of those discharged for organ donation or end-of-life care on mortality rates has not, to our knowledge, been previously explored. There is a possibility that this group exert a powerful effect on post-ICU mortality rate, especially if disproportionately discharged out-of-hours. In the authors’ experience, organ donation procedures frequently happen out-of-hours. The National Organ Retrieval Service’s annual report for 2017–18 shows markedly greater mobilisation of organ retrieval teams overnight nationally, which is consistent with this^22^.

Previous studies have considered factors after ICU discharge as potential contributors to the finding of excess mortality^12,13^. Suggestions have included patient-related factors such as severity of illness, external factors, or factors related to care in the final stages of an ICU admission including frequency of medical reviews or observations in patients who have been deemed ready for ward discharge prior to a bed becoming available. A unifying explanation for the excess out-of-hours mortality reported previously in studies has, however, not yet been identified^12,13^. Our study supports the hypothesis that reported excess mortality following out-of-hours ICU discharge may relate more to the complex hour-to-hour functioning of a modern health system than to any intrinsic failure in care^12,13^. Comparable readmission rates between those discharged in-hours and out-of-hours could support the idea that patients discharged out-of-hours were not more unwell than those discharged in-hours.

The finding that patients discharged out-of-hours had higher severity of illness, as quantified by APACHE II score, is of great interest. The recorded APACHE II score is based on physiological status at the time of admission. There is no clear explanation for why patients more unwell at the time of ICU admission were disproportionately discharged out-of-hours, in many cases several days later. The suggestion that the most unwell patients (albeit at time of admission) go on to be discharged out-of-hours is likely to be of some concern. If these patients are at all predisposed to higher mortality risk then post-ICU mortality rates may also be influenced, as the APACHE II prediction is for hospital discharge mortality rather than just ICU mortality. The finding of a comparable mortality rate, despite the out-of-hours discharge patients being more unwell on admission to ICU (demonstrated by higher APACHE II score), lends weight to our findings. Higher severity of illness in those discharged out-of-hours has been reported in a number of other studies^1,4–6,8,11,15^. Again, the explanation may relate to hospital processes with earlier discharges being elective (and therefore mainly surgical) patients for whom discharge is anticipated and more easily planned. Medical and emergency surgical patients are likely to be more unwell on admission and will not have an advanced discharge plan in the system. They may be identified as fit for discharge earlier in the working day, but leave the ICU only when a ward bed becomes available. In addition to this, pressures on ICU bed capacity resulting from emergency admissions may lead to late decisions about discharge for some patients. In-hours, the majority of patients discharged were under the care of a surgical team and out-of-hours the majority of patients were under the care of a medical team. Patients who have been identified as no longer needing critical care interventions, but who would remain longer for observation if ICU beds were abundant, may be vulnerable to out-of-hours discharge if other patients require emergency admission for organ support.

Median length of stay was longer in those discharged out-of-hours. This could relate to higher severity of illness, perhaps necessitating longer ICU stay, but the difference is relatively small in real terms. The longer delay to discharge in those discharged out-of-hours is not entirely unexpected; decisions on readiness for discharge in the authors’ experience tend to be taken during core daytime hours. Those discharged out-of-hours will therefore typically have waited longer from this point than those discharged in-hours. Once again this appears to be a hospital process finding rather than a real patient difference.

Differing definitions of the in-hours and out-of-hours periods between studies conducted in this field make direct comparisons impossible.

Our findings would be best explored further by a large multi-centre study, investigating post-ICU outcomes for patients from a broad range of medical and surgical specialties. If the results of this study are replicated, questions may be raised regarding the extent to which ICU care, patient factors and the discharge environment each contribute to post-ICU outcomes. This may prompt wider discussions about the mechanisms through which a hospital functions (particularly out-of-hours), the growing role of critical care outreach teams and ways of optimising safety of ward discharges.

Overall, in this single-centre retrospective cohort study we report no meaningful difference in in-hospital mortality rate or ICU readmission rate between those discharged in-hours and out-of-hours from the ICU, after exclusion of those discharged for organ donation or end-of-life care. Prior to this exclusion, consistent with previous studies, a difference in mortality was apparent. This analysis presents a potential alternative explanation for reported excess mortality following out-of-hours ICU discharge, related to the inclusion of organ donation and end-of-life care patients in data sets rather than standards of care delivered out-of-hours. Whilst existing literature supports excess mortality following ICU discharge out-of-hours, we are not aware of any studies investigating the influence of this patient group previously.
